# Microbiome Study of Initial Gut Microbiota from Newborn Infants to Children Reveals that Diet Determines Its Compositional Development

**DOI:** 10.4014/jmb.2002.02042

**Published:** 2020-04-09

**Authors:** Hye-Jin Ku, You-Tae Kim, Ju-Hoon Lee

**Affiliations:** Department of Food Science and Biotechnology, Graduate School of Biotechnology, Kyung Hee University, Yongin 17104, Republic of Korea

**Keywords:** Microbiome, initial gut microbiota, breast milk feeding, formula milk feeding, diet

## Abstract

To understand the formation of initial gut microbiota, three initial fecal samples were collected from two groups of two breast milk-fed (BM1) and seven formula milk–fed (FM1) infants, and the compositional changes in gut microbiota were determined using metagenomics. Compositional change analysis during week one showed that *Bifidobacterium* increased from the first to the third fecal samples in the BM1 group (1.3% to 35.1%), while *Klebsiella* and *Serratia* were detected in the third fecal sample of the FM1 group (4.4% and 34.2%, respectively), suggesting the beneficial effect of breast milk intake. To further understand the compositional changes during progression from infancy to childhood (i.e., from three weeks to five years of age), additional fecal samples were collected from four groups of two breast milk–fed infants (BM2), one formula milk–fed toddler (FM2), three weaning food–fed toddlers (WF), and three solid food–fed children (SF). Subsequent compositional change analysis and principal coordinates analysis (PCoA) revealed that the composition of the gut microbiota changed from an infant-like composition to an adult-like one in conjunction with dietary changes. Interestingly, overall gut microbiota composition analyses during the period of progression from infancy to childhood suggested increasing complexity of gut microbiota as well as emergence of a new species of bacteria capable of digesting complex carbohydrates in WF and SF groups, substantiating that diet type is a key factor in determining the composition of gut microbiota. Consequently, this study may be useful as a guide to understanding the development of initial gut microbiota based on diet.

The gastrointestinal tract (GIT) of newborn infants is rapidly colonized by members of a variety of bacterial genera after birth [1, 2]. It has been generally reported that the gut microbiota performs important functions for the human host, including protecting the infant’s intestinal health by immune stimulation [3] and preventing infantile diarrhea [4]. Previous studies have suggested that the phylum *Actinobacteria* including *Bifidobacterium* is generally dominant in breast milk–fed infants, whereas the phylum *Proteobacteria* including *Escherichia coli* is dominant in formula-fed infants [5, 6], suggesting that human breast milk may influence the composition of gut microbiota in infants. However, little research into the formation and development of initial gut microbiota in infants has been conducted yet. In addition, the compositional conversion and progression from initial infantile gut microbiota to an adult-like one in children remain inadequately understood. Therefore, it is necessary to elucidate initial formation of gut microbiota in infants and the developmental mechanism of progression to one that is adult-like.

To characterize the composition and change of gut microbiota, fecal samples were obtained from newborn Korean infants who underwent a natural delivery and children up to five years old with an approval number (KHGIRB-19-192) from the Kyung Hee University Institutional Review Board (IRB). The total fecal DNA of each sample was extracted and purified following a previous protocol [7]. The 16S rRNA genes were PCR-amplified using a 926F/1505R primer set for GS-FLX+ pyrosequencing [7] or a 341F and 805R primer set for Illumina MiSeq sequencing (Illumina, USA) [8]. The PCR products were sequenced using one of these next-generation sequencing methods. These 16S rRNA sequencing reads were analyzed using the SILVA 16S v128 database [9] for bacterial taxonomic assignments and the Microbial Genomics Module of the CLC Genomics Workbench software (Qiagen, Germany), following the default workflow and parameters for bacterial composition analysis.

For compositional analysis of initial gut microbiota in newborn infants, meconium and fecal samples were collected from two breast milk–fed (BM1) and seven formula milk–fed (FM1) infants (Table 1). Most of the identified gut bacteria in BM1 and FM1 samples belong to four major phyla: *Proteobacteria*, *Bacteroidetes*, *Firmicutes*, and *Actinobacteria* (Fig. 1A). However, microbial succession was variable. In the BM1 group, *Firmicutes* (3.16%–17.90%) and *Actinobacteria* (1.09%–31.19%) increased but *Bacteriodetes* (33.49%–1.41%) decreased (Fig. 1A). However, in the FM1 group, *Proteobacteria* (5.90%–41.53%) increased but *Firmicutes* (51.99%–26.58%) decreased. At the genus level, this trend was supported by the observed increase in *Bifidobacterium* and *Streptococcus* but decrease in *Bacteroides* in the BM1 group and the observed increase in *Serratia* but decrease in *Streptococcus* in the FM1 group (Fig. 1B). This result suggests that the different microbial succession profiles between the BM1 and FM1 groups may be due to different feeding types. In particular, a rapid increase of *Bifidobacterium* in the BM1 group may be induced by human milk oligosaccharides (HMOs) in breast milk, suggesting that breastfeeding induces beneficial maturation of gut microbiota [10]. Human breast milk differs from cow milk as it contains sialylated and fucosylated oligosaccharides, which promote specific growth of *Bifidobacterium* [11, 12]. In addition, the FM1 group exhibited a more diverse microbiota including potential pathogens relative to the BM1 group, supporting the beneficial effect of breast milk. This increase in microbiota diversity of the FM1 group probably depends upon the contents of the formula milk.

To further understand the compositional changes during development of infants to children (i.e., from three weeks to five years of age), 27 fecal samples were collected from four groups of two breast milk–fed infants (BM2), one formula milk–fed toddler (FM2), three weaning food–fed toddlers (WF), and three solid food–fed children (SF). At the phylum level, each group presents a unique major phyla pattern. In fecal samples of BM2 group, *Firmicutes*, *Proteobacteria*, and *Actinobacteria* were the major phyla identified, consistent with the similar composition of the 3rd fecal samples in BM1 (Figs. 1A and 2A). However, the fecal samples of the FM2 group revealed a low composition of *Actinobacteria*, probably due to lack of HMOs in formula milk. This trend of reduced *Actinobacteria* was also observed in the WF group. According to increasing age of children, from BM2 to SF, *Actinobacteria* decreased but *Firmicutes* increased (Fig. 2A). In addition, *Bacteroidetes* became one of the major phyla in the SF group, suggesting that the composition of the gut microbiota in the SF group was altered from the initial infant gut microbiota to an adult-like one. Therefore, this feeding type may be responsible for the compositional changes in and progression of the gut microbiota from infancy to childhood. Subsequent PCoA analysis of these compositions of the gut microbiota showed that each group (i.e., BM2, FM2, WF, and SF) was located at a unique position relative to the others in the plot, supporting the above conjecture (Fig. 2B). Interestingly, the rapid dietary change of breast milk feeding (DH1 and DH2) to weaning-food feeding (DH3) in toddler M showed re-localization in the plot from the BM2 group to the WF group (Fig. 2B). Similarly, the rapid dietary change of weaning-food feeding (JH1) to solid-food feeding (JH2 and JH3) in child Q revealed re- localization from the WF group to the SF group, suggesting that only one dietary change could modulate the overall composition of the gut microbiota. Therefore, among the factors affecting gut microbiota in the growth stage, dietary changes may be the most significant factor.

To further understand the overall compositional changes in the gut microbiota during aging from infancy to five years of age, all compositional analysis results were merged. Then, the merged data were grouped according to feeding type as breast milk feeding (BM), formula-milk feeding (FM), weaning-food feeding (WF), and solid-food feeding (SF). Compared with the compositions of the gut microbiota in the BM and FM groups, the composition of gut microbiota became more complicated and new genera of bacteria including *Alistipes, Dialister*, *Prevotella*, *Faecalibacterium*, *Ruminococcus*, *Roseburia*, and *Eubacterium* were present in the WF and SF groups (Fig. 3A). To characterize the gut microbiota associated with dietary changes, the top 15 major bacterial taxa were selected, and their proportional changes were monitored (Fig. 3B). High proportions of *Escherichia–Shigella* and *Bifidobacterium* in BM and *Streptococcus* and *Veillonella* in FM gradually decreased in the WF group, finally reaching a proportion of less than 10% in the SF group. Increased proportions of *Faecalibacterium*, *Ruminococcus*, *Roseburia*, and *Eubacterium* were observed in the WF group. Finally, *Prevotella*, *Akkermansia*, *Alistipes*, and *Dialister* were colonized as major gut bacteria in the SF group (Fig. 3B). Therefore, it is possible that these newly emerged gut bacteria may be linked to intake of various nutrients as well as dietary fiber in children eating weaning or solid foods.

Previously, it was reported that solid food intake causes bile acid production by gut bacteria to regulate the balance of the gut microbiota [13]. In the SF group, a new genus of bile-tolerant bacterium, *Alistipes*, emerged and became one of the major gut bacteria, supporting this finding. Among the new bacteria, *Dialister* became a major gut bacterium, probably due to its ability to degrade host-indigestible complex carbohydrates or dietary fiber [14]. Other new bacteria such as *Roseburia*, *Faecalibacterium*, and *Ruminococcus* also show digestive abilities that survive under nondigestible dietary fiber–rich environments prompted by ingestion of solid foods and even produce butyrate as one of the major metabolites associated with regulation and balancing of gut microbiota [14– 16]. These newly emerged gut bacteria were observed in the healthy adult gut bacterial community, showing that maturation of the gut bacterial community to an adult-like configuration is facilitated by ingestion of solid foods [17].

Consequently, this study revealed the initiation and development of gut microbiota during progression from infancy to five years of age in Korean children. Therefore, it may provide a basis for a more comprehensive understanding of the maturation of the Korean initial gut microbiota and the role of the gut microbiota in lifelong health.

## Figures and Tables

**Fig. 1:**
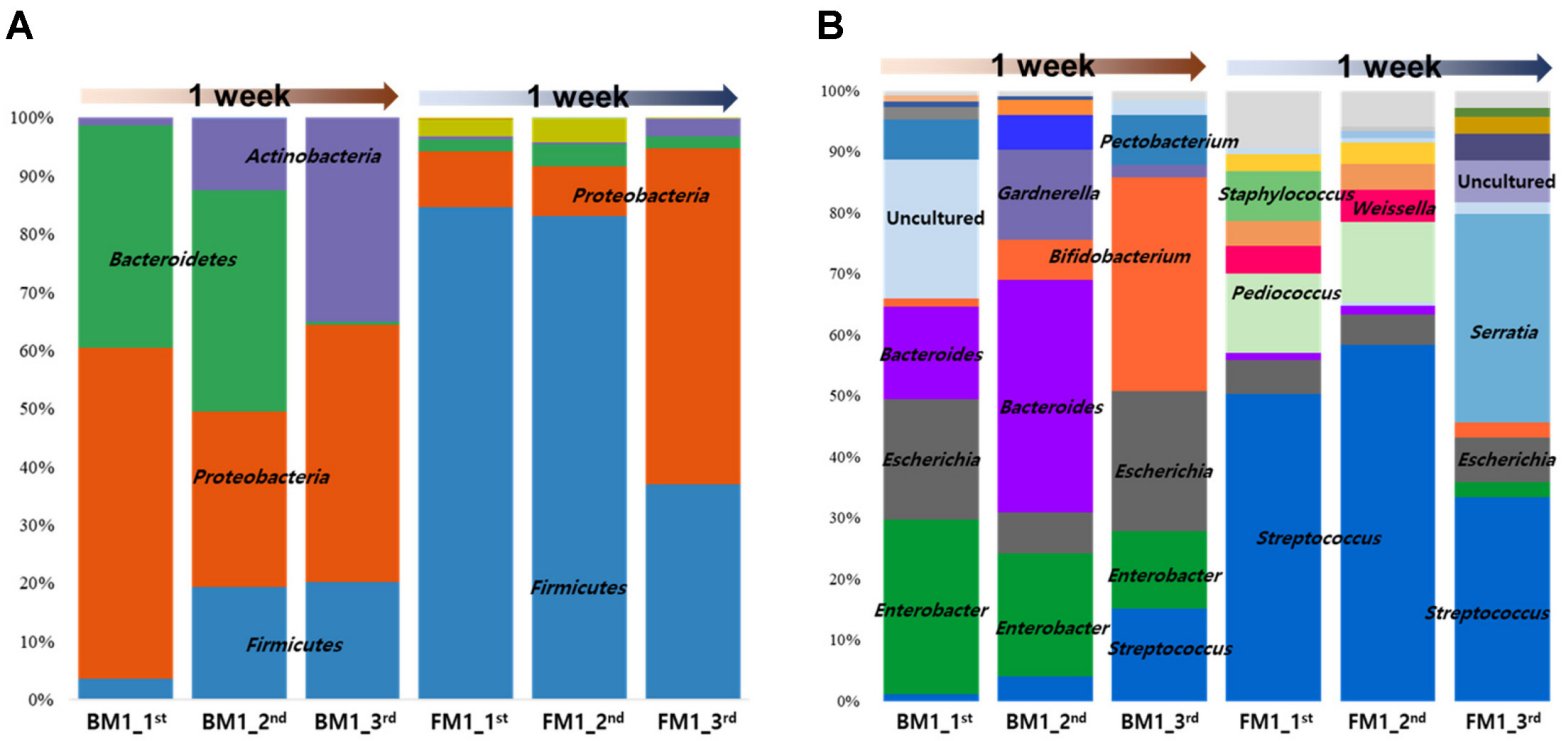
Average compositional change in gut microbiota from birth to one week of age at the phylum (A) and genus levels (B). BM1, breast milk–fed infants; FM1, formula milk–fed infants. (BM1_1^st^ contains A1 and B1; BM1_2^nd^ contains A2 and B2; BM1_3^rd^ contains A3 and B3; FM1_1^st^ contains C1, D1, E1, and F1; FM1_2^nd^ contains C2, D2, E2, and F2; FM1_3^rd^ contains C3, D3, E3, F3, G3, H3, and I3).

**Fig. 2:**
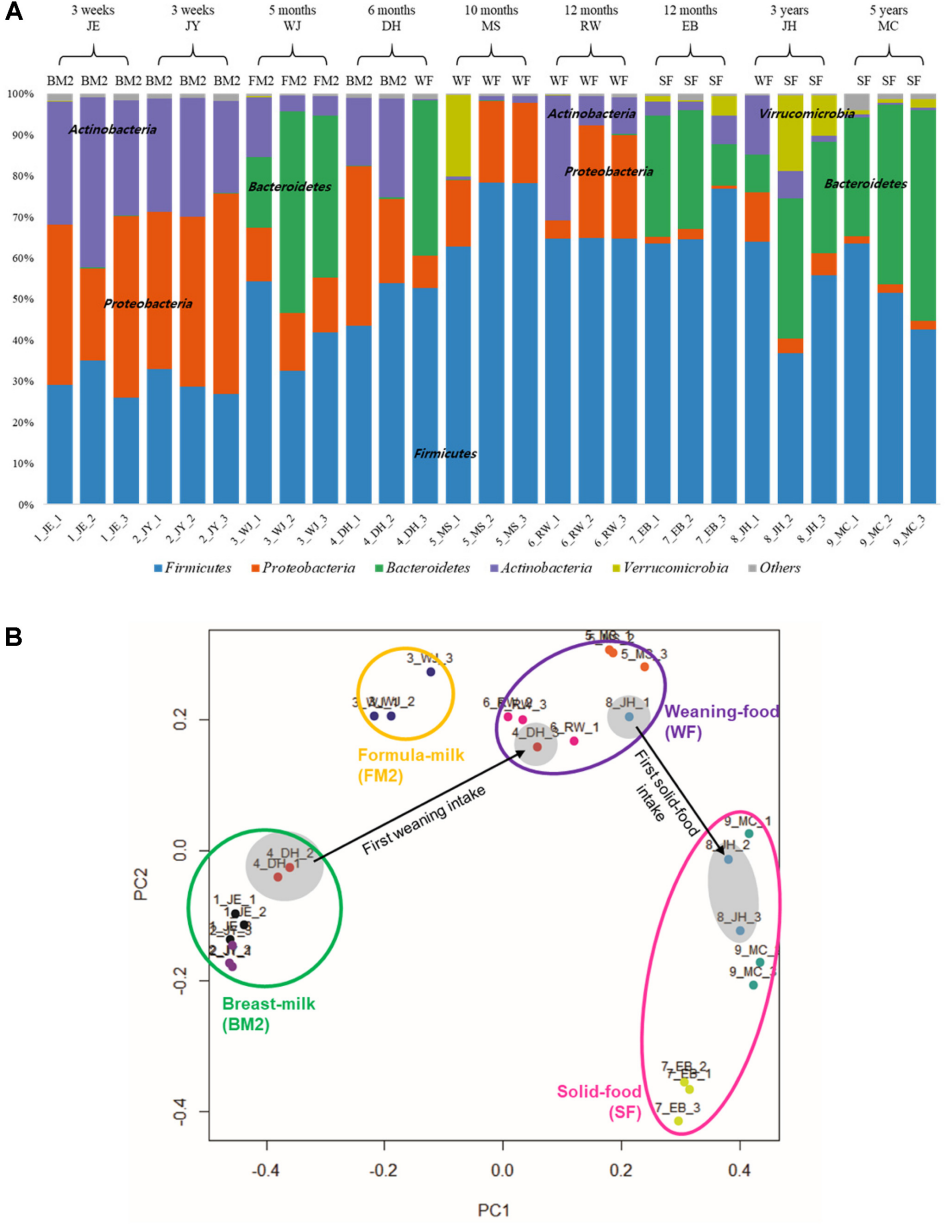
Compositional change in gut microbiota from three weeks to five years of age for evaluating the impact of diet and individual variations. (**A**) The relative abundance of the taxonomic distributions in each individual sample. BM2, breast milk–fed infant (older than one week); FM2, formula milk–fed toddler (older than five months); WF, weaning food–fed toddler; SF, solid food–fed child. (**B**) PCoA (using Bray–Curtis matrix) plot from 27 fecal samples (obtained from nine donors). The figure represents sample ID and color code based on donor. Arrows indicate changes in gut microbiota according to dietary changes.

**Fig. 3:**
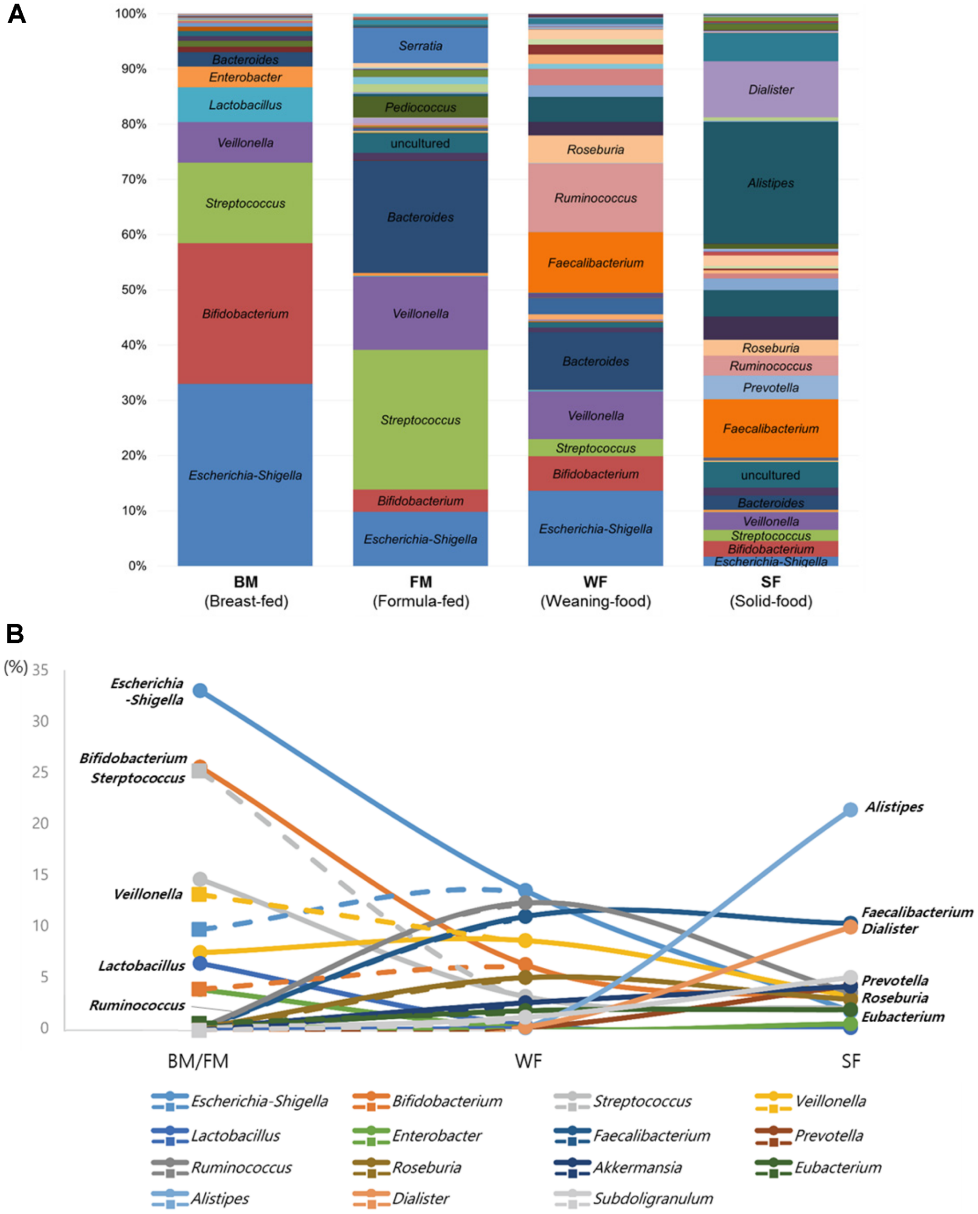
Average compositional change in overall gut microbiota from infancy to childhood. (**A**) The relative abundance of each feeding type. (**B**) Compositional gut microbiota change in 15 major bacterial taxa by dietary change. Closed circle with a solid line, BM; closed square with dashed line, FM.

**Table 1:** List of volunteers for collection of fecal samples.

Subject	Sex	Age	Group^a^	Number of samples	Sample ID	Analysis method
Newborn A	F	Newborn	BM1	Three samples collected	A1/A2/A3	454 pyrosequencing (GS FLX+)
Newborn B	F	Newborn	BM1	at one week after birth	B1/B2/B3	
Newborn C	F	Newborn	FM1		C1/C2/C3	
Newborn D	F	Newborn	FM1		D1/D2/D3	
Newborn E	M	Newborn	FM1		E1/E2/E3	
Newborn F	F	Newborn	FM1		F1/F2/F3	
Newborn G	M	Newborn	FM1	Single sample collected	G3	
Newborn H	M	Newborn	FM1	at one week after birth	H3	
Newborn I	M	Newborn	FM1		I3	
Infant J	F	3 weeks	BM2	Three samples collected	JE1/2/3	Illumina (MiSeq)
Infant K	F	3 weeks	BM2	at one-month intervals	JY1/2/3	
Toddler L	M	5 months	FM2		WJ1/2/3	
Toddler M	M	6 months	BM2→WF		DH1/2/3^b^	
Toddler N	F	10 months	WF		MS1/2/3	
Toddler O	M	12 months	WF		RW1/2/3	
Toddler P	F	17 months	SF		EB1/2/3	
Child Q	M	3 years	WF→SF		JH1^b^ /2/3	
Child R	F	5 years	SF		MC1/2/3	

a BM1, breast milk–fed infant (up to one week old); FM1, formula milk–fed infant (up to one week old); BM2, breast milk–fed infant (older than one week); FM2, formula milk–fed toddler (older than five months); WF, weaning food–fed toddler; SF, solid food–fed child.

b DH3 was sampled at the time at which the diet changed from BM2 to WF, while JH1 was sampled during WF feeding.
